# Use of failure mode and effect analysis to reduce patient safety risks in purchasing prescription drugs from online pharmacies in China

**DOI:** 10.3389/fmed.2022.913214

**Published:** 2022-07-19

**Authors:** Qinyuan Hu, Haiyao Hu, Ming Hu, Jun Zhang, Liangwen Gou, Shuping Shi, Jingyi Zhou, Naitong Zhou, Zhen Huang

**Affiliations:** ^1^Key Laboratory of Drug-Targeting and Drug Delivery System of the Education Ministry and Sichuan Province, Sichuan Engineering Laboratory for Plant-Sourced Drug and Sichuan Research Center for Drug Precision Industrial Technology, West China School of Pharmacy, Sichuan University, Chengdu, China; ^2^Administration for Market Regulation of Qionglai, Chengdu, China; ^3^West China School of Medicine, Sichuan University, Chengdu, China; ^4^Administration for Market Regulation of Chengdu, Chengdu, China

**Keywords:** FMEA analysis, prescription drugs, online pharmacy, patient safety, risk reduction

## Abstract

**Background:**

Online pharmacies have gradually penetrated the market, but pose risks to patients' health. Failure Mode and Effect Analysis (FMEA) is an effective and reliable method for reducing pharmacy and medication risks. The purpose of this study was to conduct a prospective risk analysis of the process of purchasing prescription drugs from online pharmacies in China to guarantee drug quality and patient safety.

**Methods:**

The FMEA was performed at Sichuan University, China. A multidisciplinary team was assembled comprising a leader, four regulators, four pharmacists, two experts, etc. The process was composed of eight subprocesses: searching for prescription drugs, submitting medication requirements, completing patient information forms, dispensing, delivering, etc. Brainstorming was used to identify and prioritize failure modes, propose corrective actions, and reduce risks. Risk priority numbers were the main criterion and were obtained by multiplying three scores: severity, occurrence and detectability, which were scored by the team The team proposed corrective actions for each selected failure mode.

**Results:**

A total of forty-one potential failure modes were identified, and the causes, effects, and corrective actions of the 30 top failure modes were analyzed. The highest risk value was assigned to “photocopies of paper prescriptions uploaded were reused by patients.” Three failure modes for the S value of 5 were: “drugs are eroded and polluted by moisture or insects in the process of transportation,” “the qualification information of the pharmacies were absent or fake,” and “pharmacists fail to check prescriptions in accordance with Prescription Administrative Regulation.” Of the top failure modes, 36.67% were from Step 5, delivering the drug. After taking corrective measures to control risks, the risks reduced by 69.26%.

**Conclusion:**

The results of this study proves that the FMEA is a valuable tool for identifying and prioritizing the risks inherent in online pharmacies. This study shows that there are many potential risks in the process of purchasing prescription drugs from online pharmacies, especially in the drug delivery stage. Enhanced training and the introduction of smart devices may minimize risks. Online pharmacies and Chinese regulators should consider these findings for risk mitigation and the improvement of regulations pertaining to online pharmacies.

## Introduction

Online pharmacies have gradually penetrated the market given their 24-h access, enhanced privacy, easier price comparisons, home delivery, and direct medication availability (1). According to the latest statistics released by China's Ministry of Commerce, the total sales volume of online pharmacies reached 6.70 billion yuan in 2020, accounting for 3.80% of the total sales volume of pharmaceutical e-commerce (2). Online pharmacies had 49.53 million active users in 2020, with an average customer unit price of 229 yuan and an average customer number of 11 items (2).

The COVID-19 outbreak, not only fostered patients' habit of buying drugs online, but also promoted online medical treatment (3).Thus far, China has built more than 1,100 Internet hospitals supported by public hospitals (4). Patients can obtain prescriptions through online consultations and transfer these prescriptions to online pharmacies through an internal system. Telehealth is a medical service model similar to Internet hospitals in China, that encompasses a variety of telecommunications technologies and tactics to provide remote health services (5, 6). Telehealth has been defined in all states in the US, while most countries in the EU have no formal definition of telemedicine services. In Japan, access to telehealth clinical functions was restricted to health consultations, and only during the COVID-19 pandemic did the government allow patients to receive medical care and receive prescriptions *via* the Internet (5).

However, despite the convenience and benefits, the rise of online pharmacies has resulted in increased risks to patients and greater challenges to the government. Catastrophic events caused by medication errors have occurred through online pharmacies in China. For example, the online dispensing of colchicine tablets has resulted in multiple fatalities from overdoses (7). Besides, studies have reported other irregularities, such as the sale of prescription drugs without the need to provide a valid prescription (8–13), poor traceability (13), counterfeit medicine sources (14), poor drug quality compared to conventional pharmacy-purchased products (13), e-pharmacies without a regulatory seal/logo (11, 12), lack of a precise location (15), no declaration of side effects (15), no enquiries about allergies (10), and network data security (16).

In order to regulate the development of online pharmacies and ensure confident transactions among customers, the General Pharmaceutical Council in the United Kingdom (GPhC) designed a “registered pharmacy” logo, which detailed a set of model Internet pharmacy standards that could be checked against a list of registered pharmacies and pharmacists by clicking on a link. To obtain the logo, pharmacies must meet the GPhC guidelines related to staff requirements, record keeping, pharmacy services and so forth (17). In the United States, the National Association of Boards of Pharmacy (NABP), the organization that represents all the states' boards of pharmacy organized programs called Verified Internet Pharmacy Practice Sites in 1993 and “pharmacy” program in 2014, both websites display a list of the accredited pharmacies (8, 18). Another private certification agency in the US is LegitScript, whose certifying standards have been endorsed by NABP (18). The Canadian International Pharmacy Association (CIPA), a trade association of licensed, retail Canadian pharmacies, established certification to verify that Canadian online pharmacies comply with Canadian laws (19). It was not until December 2013 that Japan opened online drug sale, and limited it to some OTC drugs, and imposed strict access control on enterprises engaged in online drug sale (20). In India, various laws such as the Information Technology Act, 2000; the Drug and Cosmetics Act, 1940; Drugs and Cosmetic Rules, 1945; Pharmacy Act, 1948; and the Indian Medical Act, 1956, govern the online pharmacies (21). At present in China, due to the immature online pharmacy market and the lack of appropriate judgment by patients, it is difficult to guarantee the drug safety of online patients. Given this situation, the establishment of a standard for online pharmacies is considered urgent.

Accordingly, it is of vital importance to reduce risk proactively. Failure Mode and Effect Analysis (FMEA), a valuable prospective analysis that incorporates methods for identifying failure modes, and their causes and effects (22), was first used in the aerospace industry in the mid-1960 s (23). It has since been revised and applied to healthcare by the Joint Commission on Accreditation of Healthcare Organizations in 2001, which has been renamed the Healthcare Failure Mode and Effect Analysis (HFMEA) (23). In 2006, based on the HFMEA, the Institute for Safe Medication Practices Canada (ISMP Canada) developed an FMEA framework that could be applied to all healthcare processes, medication use, patient identification, specimen labeling, etc. (24). ISMP Canada then applied the FMEA to pharmacy practice, proving that the FMEA was an effective and reliable method to proactively examine complex processes in this field and could be used to highlight the high-risk subprocesses that required targeting to minimize future failures and, consequently, improve patient safety (25). Several studies have proved that the FMEA is useful for the reduction of risks related to pharmacies and medication (26–32), however, no such study has been conducted for online pharmacies.

The purpose of this study, therefore, was to conduct a prospective risk analysis of purchasing prescription drugs from online pharmacies in China, not only to identify, quantify, and prioritize potential failure modes, but also to define adequate measures for risk reduction to provide suggestions for the standard of online pharmacy.

## Method

### Study design

The FMEA was performed at Sichuan University, China, from June 2020 to January 2021. The failure mode and effect analysis followed a stepwise approach developed by ISMP Canada (25, 33).

### FMEA steps

#### Process selection and team assembly

Given that this study focused on the characteristics of online pharmacies, we assumed that the quality of drugs met the expected standards in the process of transporting the drugs from the manufacturer to the online pharmacies. Thus, we chose to examine the next process by which drugs are transported from online pharmacies to patients.

To create a high performing team, we considered the risk management tips proposed by the American Society for Healthcare Risk Management (ASHRP) and ISMP Canada. ASHRP suggested that the FMEA team should consist of a subject matter expert(s), a leader, a facilitator who understands the FMEA process, and a neutral party whose perspective would be helpful in thinking outside of the box (23). ISMP Canada suggested that the FMEA team should consist of front-line practitioners and management, as they have a clear understanding of the details and challenges of the day-to-day work as well as a perspective on resource management (33).

A 13-member multidisciplinary team comprising a leader who had experience guiding an FMEA team and was familiar with drug supply chain regulations in China, played a major role in the process. Subject matter experts included four drug circulation regulators; four online pharmacy pharmacists, two of which had middle-level management experience and two of which had over 20 years' experience in drug supply chain; two professors of drug policy, and one member of the Association of Pharmaceutical Commerce in Chengdu. Also included was a patient representative in the role of a neutral party who was not intimately familiar with the process, but whose perspective would be helpful to thinking outside of the box (23, 33). Apart from the patient, all team members were familiar with the drug supply chain and the administration of online pharmacies, and thus capable of proposing steps toward systemic risks mitigation and potential corrective actions. During the process, the team members met five times for 2 h per meeting.

#### Mapping the process

Based on prior selection method criteria, we chose isotretinoin for our preliminary study (34). Isotretinoin is a prescription medication used to treat severe recalcitrant nodular acne, which is known to cause birth defects, depression, and suicidal thoughts (35). The FDA required that it be approved for marketing only under the iPLEDGE REMS risk evaluation and mitigation strategy restricted to healthcare providers, designees, and pharmacies, to minimize fetal exposure (35, 36). In China, the pharmaceutical forms of isotretinon available include soft capsules and gels. The oral administration of isotretinon is a major systemic treatment for acne (37, 38). Among the commonly used acne drugs in China, isotretinic acid had the largest market share in 2019 (39). At present, China's risk management and adverse reaction information notification of isotretinon is based on assessments of other countries' regulatory regimes, without mentioning more detailed risk control measures (40).

We searched for “isotretinoin” on the five most popular platforms in China—Ali Health, Meituan, Jingdong Health, Dingdang, and Jianke (41)—to preliminarily identify the process by which online pharmacies sell prescription drugs. Ali Health, Meituan, Jingdong Health are online vendor sites for various products—they are both third-party platforms and have established their own online pharmacies. Dingdang and Jianke are Internet pharmacies, and only provide drugs. The team discussed and revised the process and further determined the subprocesses.

#### Identification of potential failure modes, causes, and effects

Based on the confirmed subprocesses, the team brainstormed the various aspects that could potentially go wrong in each subprocess. Then, the failure modes were discussed and finalized. Next, the team analyzed the causes of the failure modes from the perspectives of manpower, machine, material, method, and environment. The effects discussed mainly referred to the influence on drug quality and patient medication.

#### Prioritization of failure modes

The prioritization process was achieved by assigning a risk priority number (RPN) to each failure mode. The RPN was obtained by multiplying three scores: S, the severity of the outcome; O, the frequency of occurrence; and D, the likelihood of detection of the failure before the effect becomes evident (RPN = S × O × D). O could refer, not only to the data from previous adverse events, but also to the personal experience of the team members (42). We used a 5-point scale to score S, O, and D, created by combining two pre-defined scales (Table 1) (32). The final results were expressed as the median values for S, O, and D of each failure mode.

**Table 1:** Ranking scale for failure modes' severity, occurrence and detectability (32).

**Score**	**Rating scales**	**Score assigned rules**
**Severity**		
1	No effect	Failure affecting neither the patient nor the process
2	Minor	Failure causing minor effect or perceived as a nuisance to the patient or process, without causing any injury or requiring an increase in the level of health care
3	Major	Failure causing some performance loss, which can potentially necessitate an increased level of health care provided to the patient, requiring hospitalization or extending the length of hospital stay
4	Critical	Failure causing a high degree of performance loss, having a permanent impact on the patient, resulting in reduced functioning; surgical intervention may be necessary
5	Catastrophic	Failure causing deadly outcome or major, permanent loss of function
**Occurrence**		
1	Remote	1 case in 10,000 patients
2	Low	1 case in 5,000 patients
3	Moderate	1 case in 2000 patients
4	High	1 case in 100 patients
5	Very high	1 case in 20 patients
**Detectability**		
1	Remote	0 times out of 10
2	Low	2 times out of 10
3	Moderate	5 times out of 10
4	High	7 times out of 10
5	Very high	9 times out of 10

The failure modes were sorted according to the RPN, with greater RPN highlighting greater risk. According to the ISMP guidelines, we chose 70% as the pre-defined cut-off value (33). This meant that 70% of the identified failure modes with the highest criticality scores were further processed. Data analysis was conducted using Microsoft Office Excel 2016.

#### Development of corrective actions and risk reassessment

The team proposed corrective actions for each selected failure mode. To test the validity of the actions, the S, O, and D scores were reevaluated and the RPNs of failure modes were recomputed.

## Results

**Figure 1:**
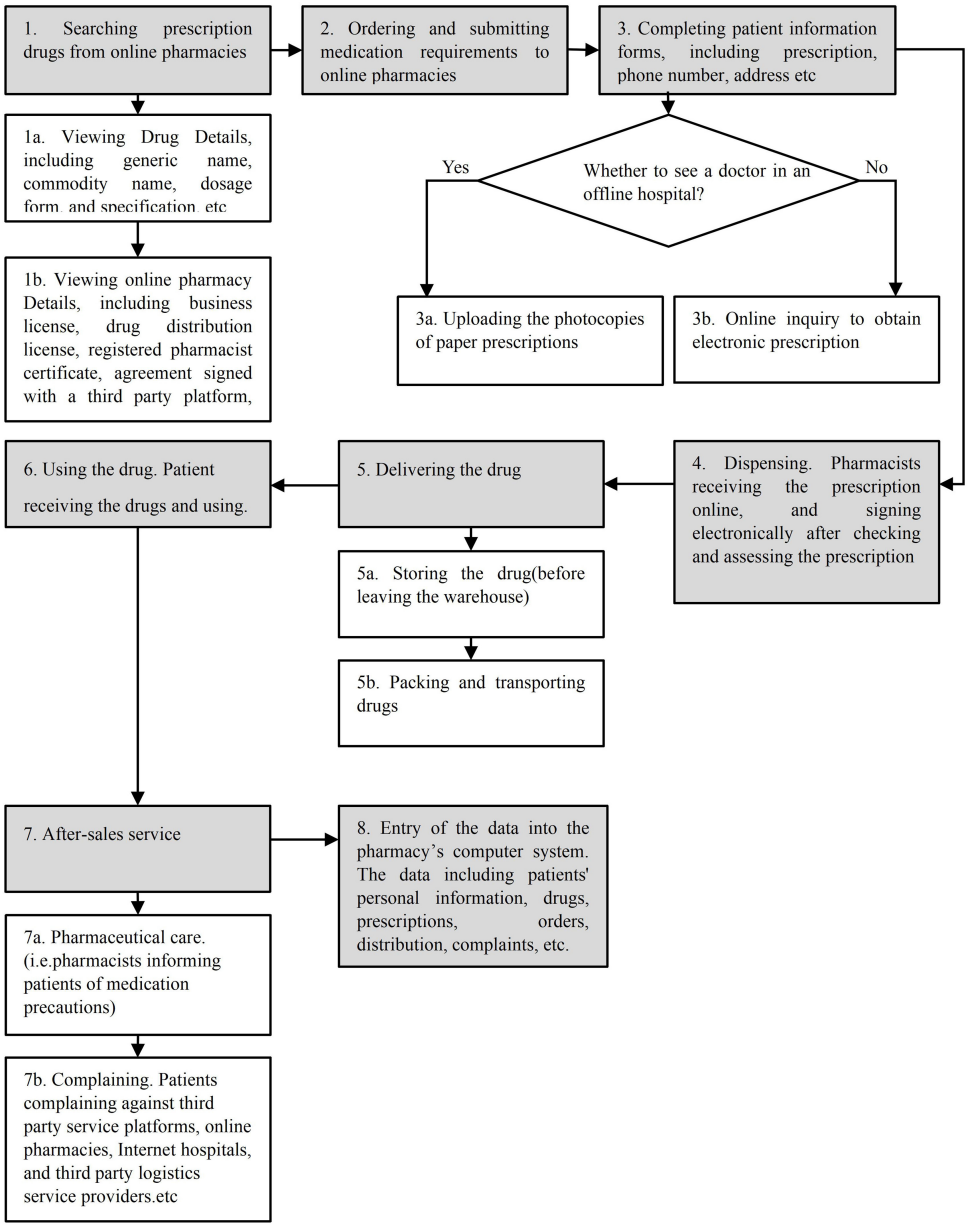
Flow chart of the purchasing process for prescription drugs from online pharmacies.

The process of purchasing prescription drugs from online pharmacies consisted of eight major steps and eight subprocesses, as shown in Figure 1. The entire process yielded a total of 41 failure modes and 87 causes. The value of RPNs ranged from 64 to 18.

### The FMEA of the process

#### Failure modes

According to the predefined cut-off value, the first 30 failure modes were selected for further processing (41^*^70%≈30). Their individual RPN values are summarized in Table 2, with the sum of RPNs amounting to 1,158. The highest risk failure mode was: “photocopies of paper prescriptions uploaded were reused by patients” (RPN 64). There were eight second-highest risk failure modes (RPN 48), including: “confounding the classification management of prescription drugs and non-prescription drugs for “double classification” drugs,” “the paper prescription uploaded exceeded the prescription expiration date,” and “the online consultation of doctors was replaced by questionnaires leaving the default responses in place, and patients bypassed this by following the default process without entering any patient-specific information” Three failure modes for the S value of 5 were: “drugs were eroded and polluted by moisture or insects in the process of transportation,” “the qualification information of the pharmacies were missing or fake,” and “pharmacists fail to check prescriptions in accordance with the Prescription Administrative Regulation.” Of the top failure modes, 36.67% (11/30) were from Step 5, delivering the drug and 16.67% (5/30) were from Step 1, searching prescription drugs in online pharmacies; four failure modes were from Step 3, completing patient information forms; Step 4, dispensing, and Step 7, after-sales service, while two failure modes were from Step 8, entry of the data into the pharmacy's computer system.

**Table 2:** Top critical failure modes, underlying causes, and corrective actions.

**No**.	**Process step**	**Failure modes**	**Causes**	**Effects**	**Corrective actions**	**Initial value**				**Revised value**			
						**S**	**O**	**D**	**RPN**	**S**	**O**	**D**	**RPN**
1	3a	Photocopies of paper prescriptions uploaded were reused by patients	-Patients knowingly purchased drugs in excess of prescribed limits -Online pharmacies or platforms lacked software systems to identify errors	The patient's prescription drug dosage exceeds the limit, and overmedication is harmful to health	Identifying multiple orders with the same address, account, and payment account by the computer system	4	4	4	64	3	2	2	12
2	1a	Confounding the classification management of prescription drugs and non-prescription drugs for “double classification” drugs ^*^	-Lack of awareness among online pharmacies or platforms	Misleading patients about drug use	Marking “double classification” on the web page and highlighting the indications of the drug	4	4	3	48	3	3	2	18
3	3a	The paper prescription uploaded exceeded the prescription expiration date	-Pharmacists did not strictly review prescriptions -Insufficient pharmacists' knowledge about the assessment of prescriptions -Excessive workload of pharmacists	The drugs listed in the prescription may not match the health condition of the patients, leading to the wrong medication	Training pharmacists	4	4	3	48	3	2	2	12
4	3b	The online consultation of doctors was replaced by questionnaires leaving the default responses in place, and patients bypassed this by following the default process without entering any patient-specific information.	-Online pharmacies coped with regulatory requirements -Internet hospitals were established to save doctor manpower costs -Excessive workload of Internet doctors -Platforms were negligent	Patients may not assess their true medical condition, and the collected patient information may be wrong, which could lead to drug allergy and interactions; the use of drugs by pregnant and lactating women could also affect the health of the fetus or baby	Establishing standard electronic consultation procedures. Carrying out regular training for Internet doctors to constantly improve their professional level and risk-prevention awareness	4	4	3	48	3	2	2	12
5	5b	Medicines were mixed with other deliveries (online meal ordering, etc.) and were not segregated	-Lack of separate storage areas	Medicine may be contaminated	Packaging drugs, which could prevent loose and contaminated drugs; materials should ideally be waterproof and wear resistance, such as foam paper	4	4	3	48	3	2	2	12
6	5b	Delivery people were not pharmaceutical professionals and did not understand the requirements of drug storage and distribution	-Lack of staff training	The quality of medicine may be affected	Training delivery people	4	4	3	48	3	2	2	12
7	5b	Some medicines (emulsions, etc.) deteriorate drastically during transportation due to turbulence	-No appropriate facilities and equipment -Employees did not understand the requirements of drug distribution	The quality of medicine may be affected	Training staff; using foam paper for packaging	4	4	3	48	3	2	2	12
8	4	Pharmacists are not on duty	-Lack of pharmacists -Platforms were negligent	Patients' medication problems could not be professionally solved	Introducing policies to stimulate pharmaceutical professionals to register for pharmacists to fill the gap; strictly supervising the suspension of pharmacists.	4	4	3	48	3	2	2	12
9	3a	The uploaded prescription photocopy is not the real prescription	-Pharmacists did not strictly review prescriptions -Lack of software to identify prescriptions -Prescription photos were not clear enough to be recognized -Platforms were poorly regulated	Medication errors or overdoses could affect the health of patients	Training pharmacists	4	4	3	48	3	2	2	12
10	1b	The pharmacies' qualification information was missing or fake	-Online pharmacies lacked awareness of displaying pharmacy information for patients -Online pharmacies intentionally hid pharmacy information -No regulations for negligence of those uploading information -Platforms were negligent -Those uploading information were negligent	Patients cannot judge whether or not online pharmacies are legal	Standardizing the content and standard of online pharmacy qualification information display. The content should include business license, drug distribution license, registered pharmacist certificate, agreement signed with a third party platform. Standards include completeness and clarity of the photo	5	3	3	45	3	2	2	12
11	4	Pharmacists fail to check prescriptions in accordance with the Prescription Administrative Regulation (43)	-Pharmacists were not careful -Insufficient professional competence of pharmacists -Excessive workload of pharmacists	Unreasonable prescriptions, prescription overdose, repeated administration, and other problems affect the health of patients	Training pharmacists	5	3	3	45	3	2	2	12
12	7b	No complaint window or no way of dealing with complaints	-Online pharmacies were shunning complaints -Lack of awareness among online pharmacies or platforms	Patients cannot protect their rights	Improving the complaint system. Setting up a complaint window on the page, arranging someone to deal with complaints, and summarizing the issues regularly	4	3	3	36	2	2	2	8
13	5a	The drug nearing expiry	-Online pharmacies deliberately promoted near-term drugs	Expired drugs may affect the health of patients	Proactively informing patients of drug expiration dates to ensure that patients are aware	4	3	3	36	3	3	2	18
14	5b	No system or hardware/software equipment for full traceability	-Online pharmacies lacked the awareness to establish drug traceability -Platforms were negligent	Unable to track	Developing the system for full traceability and equipping it with hardware and software	4	3	3	36	3	2	2	12
15	1b	Failed to provide real and valid contact information of pharmacies and failed to update the information in time when it changed	-Online pharmacies lacked awareness of displaying pharmacy information for patients -Online pharmacies intentionally hid pharmacy information -No regulations on negligence of those uploading information -Platforms were negligent -Those uploading information were negligence	The patient may be unable to contact the pharmacy	Formulating rules regarding the contact information provided by the pharmacy to ensure that the patient can contact the pharmacy	4	3	3	36	2	2	2	8
16	1a	Medium- and high-alert drugs were sold at online pharmacies	-Online pharmacies only sought to make profits -Employees were negligent -Platforms were negligent	Patients may abuse narcotic, psychotropic, and other specially controlled drugs, causing harm to their own health or using them for illegal purposes; medicines may deteriorate due to improper storage	Controlling the business scope of online pharmacies by prohibiting (1) vaccines; blood samples; narcotic, psychotropic, toxic, and radioactive drugs; and precursor chemicals; (2) long-term drugs that may be excessively used, producing drug dependence or seriously damaging health, such as antibiotics; (3) drugs that will not keep, such as cold-chain drugs.	4	3	3	36	3	2	2	12
17	5b	Unsuitable temperature and humidity during transportation	-Not equipped with temperature and light control equipment -Delivery personnel did not grasp the precautions of drug transportation	The quality of medicine may be affected	Equipping staff with temperature and light control equipment and training them	4	3	3	36	3	2	2	12
18	4	Chemical medicine pharmacists provided prescription review and medication guidance services for Traditional Chinese medicine (TCM)	-Lack of TCM pharmacists -Online pharmacies did not manage such situations -Platforms were negligent	Failed to accurately identify prescription problems involving TCM prescription drugs	Coordinating Chinese pharmacists and chemical medicine pharmacists, and stipulating that only Chinese medicine pharmacists can provide prescription review and medication guidance services	4	3	3	36	3	2	2	12
19	4	Prescriptions for children or elderly patients are approved	-Pharmacists were not careful -Insufficient professional — competence of pharmacists -Excessive workload of pharmacists	Children and the elderly could buy drugs by mistake	Training pharmacists	4	3	3	36	3	2	2	12
20	7a	Patients cannot inform online pharmacies of adverse drug reactions	-No channel for consumers to report adverse reactions -Lack of pharmacist—patient interaction	Patients with adverse drug reactions cannot take timely and effective treatment measures	Establishing patient reporting system to take remedial measures; providing 24-h toll-free telephone service for medication consultation	4	3	3	36	3	2	2	12
21	5b	Delivery personnel suffer from infectious diseases or other diseases that may contaminate drugs	-No physical examination	Medicine is contaminated and patients are infected	Carrying out pre-job and regular physical examinations for delivery personnel.	4	3	3	36	3	2	2	12
22	5b	The vehicle or device malfunctioned during transportation	-No inspection before transportation	Drugs deteriorate or are delayed in terms of use by patients	Stipulating that the vehicle facilities and equipment should be inspected before leaving the vehicle; making emergency plans for facility failure	4	3	3	36	3	2	2	12
23	1a	Photos of drug instructions were unclear and incomplete	-Online pharmacies tried to induce patients to buy drugs -No unified regulations for online pharmacies or platforms -The uploader was careless -Platforms were negligent	Misleading patients with medication	Ensuring that information about drug instructions are in the same font, size, and color to meet the requirements of patients for browsing and viewing drug information, including the generic name, commodity name, dosage form, and specification; arranging a review	4	4	2	32	3	2	1	6
24	5b	Drugs were eroded and polluted by moisture or insects in the process of transportation	-No box or mat isolation	Medicine may be contaminated	Equipping delivery personnel with isolation boxes or mats and training them	5	3	2	30	3	2	2	12
25	5b	The transportation distance or time was too long	-Delivery personnel were unfamiliar with distribution routes -Not properly allocating distribution routes	The quality of drugs may be affected or patients may experience delays	Introducing modern logistics system and training delivery personnel	3	3	3	27	2	2	2	8
26	5b	The drugs directly touched the floor or wall during transportation	-No box or mat isolation -Employees did not understand the properties of drugs	Medicine may be contaminated	Equipping delivery personnel with isolation boxes or mats and training them	3	3	3	27	2	2	2	8
27	7a	Shortage of pharmacists providing pharmaceutical care	-Lack of pharmacists	Patients' medication problems cannot be professionally solved	Introducing policies to stimulate pharmaceutical professionals to register for pharmacists to fill the gap; strictly supervising the suspension of pharmacists.	3	3	3	27	3	2	2	12
28	8	Data recording was untrue, untimely, incomplete, or inaccurate^**^	-Online pharmacies/employees intentionally falsified data -Employees had a weak sense of responsibility -Lack of staff training -Online pharmacies or platforms were negligent	Influences prescription authenticity, patient medication and drug quality; there is no way to verify the content of complaints	Developing uniform data recording standards and training staff	3	3	3	27	3	2	2	12
29	8	Data was leaked or storage time was too short and data such as web pages and audio/video files were damaged^**^	-The data retention time was not uniform -Online pharmacies or platforms were negligent	Influences prescription authenticity, patient medication, and drug quality; there is no way to verify the content of complaints	Developing uniform data storing standards and training staff	3	3	3	27	3	2	2	12
30	7b	Third party service platforms, online pharmacies, Internet hospitals, and third party logistics service provider shirked responsibility	-No liability agreement was reached	No one is responsible for the medication problems of patients	Signing an agreement with the platform taking the responsibility first; then, the platform shall pursue responsibilities according to their respective functions.	3	3	3	27	3	3	2	18

* *“Double classification” drugs in China can be defined as the same drugs having two legal classifications: prescription drugs and non-prescription drugs depending on the indication (44)*.

** *The data referred to in this study includes patients' personal information, drugs, prescriptions, orders, distribution, complaints*.

#### Causes

The main causes for top failure modes can be summarized as follows: (1) manpower, including lack of pharmacist—patient interaction, pharmacists and delivery personnel mainly lack training, capability, and professional pharmaceutical knowledge, and are negligent in terms of running online pharmacies or platforms; (2) machines to ensure the quality of drugs, such as temperature and humidity monitoring equipment, may not be adequate or may malfunction; (3) causes related to materials include non-standard packaging of drugs, no box or mat isolation, etc.; (4) method-related causes mainly refer to the lack of standardized and unified management, such as online pharmacy information and drug information displays; (5) finally, “environment” refers to the environment in which the failure mode occurs, such as the overall insufficiency of pharmacists (Table 2).

#### Effects

The failure mode effects can be classified as the effect on drug quality and the effect on patient medication process. From the perspective of drug quality, contaminated and expired drugs impact the effectiveness, safety, and stability of the drugs. The effects on the patient medication process refer to the fact that the failure mode may lead to the wrong drug selection and the mismedication of patients (Table 2).

#### Corrective actions

The team recommended corrective actions to overcome the abovementioned identified failure modes (Table 2). Among them, staff training is probably the most important action that could improve the pharmaceutical professional level; it includes training for both pharmacists and delivery personnel. Formulating rules is another important way to fill the loopholes of the existing process, such as specifying the content and format of drug instructions on web pages and implementing uniform provisions for electronic consultation procedures. Introducing advanced intelligent facilities, such as intelligent drug delivery equipment, modern logistics system, and prescription review system, can prevent human errors in the process. Good Supply Practice (GSP) requirements, such as providing an isolation box or mat and equipping facilities for traceability during drug transportation, should also be met.

#### Risk reduction

As shown in Table 2, after taking corrective measures to control risk, the RPN amount decreased significantly after reevaluation, from 1,158 to 356 (69.26%) in all of the top failure modes. For the highest-risk failure mode—“photocopies of paper prescriptions uploaded were reused by patients”—the RPN value recorded the greatest drop from 64 to 12. The RPN value of another failure mode with maximum risk reduction—“photos of drug instructions were unclear and incomplete”—dropped from 32 to 6. The RPN value for the minimum risk reduction failure mode—“third party service platforms, online pharmacies, Internet hospitals, and third party logistics service provider shirked responsibility”—fell from 27 to 18 (33.33%). The three failure modes with S value of 5 reduced to 3.

## Discussion

This study used FMEA to identify and prioritize the potential risks of purchasing prescription drugs from online pharmacies and to identify ways to successfully decrease these risks. From the combination of this study's findings and experience, and an ongoing monitoring program, we perfected the online pharmacy supervision mechanisms in Chengdu, China.

“Photocopies of paper prescriptions uploaded were reused by patients” was the highest risk failure mode, which was also highlighted in other studies (8, 45). This convenience kept most patients from visiting doctors, except for those with chronic diseases. However, over a long period of time, the toxicity accumulation of some drugs, such as isotretinoin, can harm the health of patients, who need to be checked for liver toxicity after several months. It is easy for patients to ignore such adverse drug reactions when they purchase drugs independently without the supervision of a doctor (46). To prevent such situations, online pharmacies should identify multiple orders with the same address, account, and payment accounts *via* their computer systems.

The following failure modes—“photocopies of paper prescriptions uploaded were reused by patients,” “the paper prescription uploaded exceeded the prescription expiration date,” “ the online consultation of doctors was replaced by questionnaires leaving the default responses in place, and patients bypassed this by following the default process without entering any patient-specific information” and “the uploaded prescription photocopy is not the real prescription”—can be classified as related to having no effective prescription, which was the most common in the existing research literature (8–13, 18, 46–48). This requires pharmacists to strictly review prescriptions. However, their excessive workload and insufficient knowledge, as well as unclear prescription photos may pose major obstacles. Online pharmacies need to strengthen the training of pharmacists to improve their ability to identify prescriptions.

The second highest failure mode is: “confounding the classification management of prescription drugs and non-prescription drugs for “double classification” drugs.” “Double classification” drugs in China can be defined as the same drugs havings two legal classifications: prescription drugs and non-prescription drugs, depending on the indication (44). Such drugs accounted for a large proportion. In the list of non-prescription drugs published by the National Medical Products Administration of China (data as of December 18, 2021), double classification drugs account for 272 out of 1,117 and 1,126 out of 3,959 non-prescription chemical drugs and non-prescription TCM, respectively (49). These drugs have a special identity and low popularity, making it difficult for patients to identify or distinguish between them. Online pharmacies should implement classified management of double classification drugs with prominent fonts, obvious labels, and clear statements, distinguishing prescription drugs from non-prescription drugs in indications, dosage forms, specifications, and other aspects.

According to the analysis of failure modes, step 5, or drug delivery, had the most failure modes. Drug delivery (including storage)—markedly different from offline drug purchases—is closely linked to the quality of drugs, which may be one of the reasons why products offered by online pharmacies may not have the same quality as those offered by a retail pharmacy (48, 50, 51). This is because the active ingredients in drugs may have undergone significant degradation due to inadequate or improper storage and/or delivery conditions (48). One study also evaluated the packaging of the drugs purchased, revealing many problems in the drug samples including loose blister packs, capsules, or tablets in clear plastic bags without labels, the lack of packaging, or packaging that had been tampered with (47). Thus, online pharmacies should introduce modern logistics systems and provide proper packaging, isolation boxes, temperature and light control equipment, and well-trained delivery personnel.

“A shortage of pharmacists to provide pharmaceutical care” has attracted international attention. NABP found no evidence for how consumers could contact the pharmacy for advice about medicines and the Pharmacy Board of Australia recognized that there were circumstances where these forms of communication were necessary or appropriate for the patient's circumstances (52). Therefore, providing medication consultation services is necessary to avoid medication errors.

Our study has two main limitations: First, the FMEA procedure solely depended on brainstorming to identify failures, the effects of failures, causes, and failure mode scoring. Hence, to some extent, the results were subjective and inaccurate. However, our team had covered as many fields as possible to minimize the influence of subjectivity. Second, Shebl et al. cast doubt on its validity due to the procedure of calculating RPN (53–55); the reliability of results may also be affected. To minimize the impact, we used 70% as the cut-off value and analyzed failure modes with high severity, regardless of the probability of occurrence. Thirdly, the subject of our study is the process by which drugs are transported from online pharmacies to patients, therefore, the conclusion of our study has certain limitations.

## Conclusion

The results of this study prove that the FMEA is a valuable tool for mapping the process, and identifying and prioritizing the potential risks for prescription drugs from online pharmacies. Causes and effects have also been analyzed to propose corrective actions to re-design the process, which have successfully decreased the RPN value of failure modes. This study shows that there are many potential risks in the process of purchasing prescription drugs from online pharmacies, especially in the drug delivery stage. These failure modes may result in drug deterioration, medication errors, etc., and ultimately harm patients' health. Chinese regulators need to consider these findings to establish online pharmacy standards to enhance the regulation of online pharmacies.

## Data availability statement

The original contributions presented in the study are included in the article/supplementary material, further inquiries can be directed to the corresponding author.

## Author contributions

QH: conceptualization, methodology, investigation, and writing—original draft. HH: writing—original draft. MH: review and editing. JZha: resources and supervision. LG: Software. SS and JZho: visualization and investigation. NZ: conceptualization, funding acquisition, resources, supervision, writing—review, and editing. ZH: conceptualization, funding acquisition, and supervision. All authors contributed to the article and approved the submitted version.

## Funding

National development and research center for licensed pharmacist of China Pharmaceutical University Topic: Research on pharmaceutical care standards provided by licensed pharmacists in social pharmacies and its value evaluation – a case study of Sichuan Province Project number: 201809.

## Conflict of interest

The authors declare that the research was conducted in the absence of any commercial or financial relationships that could be construed as a potential conflict of interest.

## Publisher's note

All claims expressed in this article are solely those of the authors and do not necessarily represent those of their affiliated organizations, or those of the publisher, the editors and the reviewers. Any product that may be evaluated in this article, or claim that may be made by its manufacturer, is not guaranteed or endorsed by the publisher.
